# An Account of the Poisonous Effects of the Oenanthe Crocata, or Hemlock Dropwort

**Published:** 1784

**Authors:** 


VI. *An account of the poiſonous effects of the Oenanthe Crocata, or Hemlock Dropwort; by* Richard Pulteney, M. D. F. R. S. *phyſician at Blandford. Communicated in a letter to* Maxwell Garthſhore, M. D. F. R. S. & S. A. *and by him to Dr.* Simmons.

A Perſon in the neighbourhood of Blandford having lately been poiſoned through a miſtake, and the circumſtances of the fact variouſly miſrepreſented, has induced me to draw up a brief ſtate of the caſe, from the beſt information I could procure after her death; and it is accompanied by ſome obſervations, which may have have a tendency to put people on their guard against ſuch errors.

It had been recommended to this poor woman to take the juice of *water parſnep* and *water creſſes.* The perſon employed to procure theſe herbs, unhappily miſtook, for the former of them, the *hemlock dropwort*; and, as an aggravation of the evil, the juice of the root, which is more virulent than that of the leaves, was uſed. She drank little, if any, more than half a tea-cup full, in the morning faſting. In leſs than a quarter of an hour ſhe complained of exceſſive giddineſs, and ſoon after vomited, but not plentifully: before the half hour was completed, convulſions came on, and ſhe was from that time deprived of the power of ſwallowing, and of her ſenſes. In this condition, owing to the conſternation the family was in, little or nothing was got down, and the fits following each other faſt, and increaſing in ſtrength, after having ſuffered nine or ten of them, ſhe died in two hours and an half from the time of ſwallowing the fatal potion.

### OBSERVATIONS.

The two moſt poiſonous plants perhaps naturally growing in England, are ſuch as have, from their general resemblance to the *water parsnep*, been frequently mistaken for it, and numerous instances have confirmed the very virulent effects of them. These two plants are the *hemlock dropwort* (oënanthe crocata), and the *long-leaved water hemlock* (cicuta virosa). These, together with the *water parsnep*, the *hemlock*, or kex, as it is vulgarly called, *smallage*, *fools parsley*, or lesser hemlock, and many others of the same natural class called by Botanists umbelliferous plants, have such a general likeness in the manner of growing, the form of the leaves, and in the mode of flowering, that it is not at all surprising, that unskilful people, and those not used to the nicer discriminations, which hold among plants of the same natural class, should mistake one for the other; add to this, that the smell of the herb, and the root of *hemlock dropwort*, not unpleasant, nor very unlike that of the parsnep, may still farther impose upon the ignorant and unwary.

*The long-leaved water hemlock* is rarely found in the county of Dorset; whereas the *hemlock dropwort* is very common, much more so than the *water parsnep*, by the sides of the rivers and brooks, and in wet ditches, particularly such as communicate with running waters, by which means means the ſeeds and roots are carried by floods to a diſtance from the borders of the rivers. It is never ſeen on dry and hilly grounds, nor even meadows, unleſs in ſuch as are liable to be flooded. The leaves are much ſmaller cut, more ſucculent, and of a deeper green, than thoſe of the *water parſnep*, and more reſemble thoſe of *ſmallage* and *parſley*. But there is one diſtinction which will never fail, if properly attended to, and it is earneſtly recommended to all thoſe who are dubious of the plant they mean to procure, to attend to it. The root of the *hemlock dropwort* conſiſts, not of one conical or top-root, like a parſnep, neither of a number of fibres, nor of a ſingle round bulb or two, but is what may be called a fingered root, made up of a number of oblong roots, all proceeding from the bottom of the ſtalk as from a center, and in proportion to the age or vigour of the plant, ſmaller or larger, each of them uſually as thick, and nearly as long, as the finger, and from three, or four, to ſix, eight, or ten in number.

Of the *water parſnep* there are three ſpecies growing in England, each of them more or leſs frequently found in different parts of the kingdom; and all growing in water or watery ſituations. Theſe are the *ſium latifolium*, broadleaved water parſnep ; *ſium anguſtifolium*, or *berula officin.*; and the *ſium nodiflorum*, creeping water parſnep. The two firſt are thoſe which were intended to be uſed in medicine, and the ſecond particularly, is that which tradition has ſo ſtrongly recommended as an *antiſcorbutic*; but, as the laſt ſpecies is the moſt common, and is more eaſily procured, it is uſually gathered for medicinal purpoſes, inſtead of the other. The miſtake is perhaps of no conſequence, as it appears to be innocent. The roots of the firſt kind are, on good authority, ſaid to be noxious; they certainly have proved ſo to brutes. The juice of the leaves of the two others may be drank to the quantity of a quarter of a pint, and is ſafe and harmleſs. It is perhaps however to be regretted, that the *water parſnep* ſhould have obtained the character of a ſpecific with many people, in what are called ſcorbutic caſes; ſince it has occaſioned ſuch fatal errors, from miſtaking for it, not only the *hemlock dropwort*, but *the long-leaved water hemlock* in many inſtances, and ſince alſo it may juſtly be doubted, whether the virtue attributed to *water parſnep*, as an *antiſcorbutic*, does not reſt, howſoever ſanctioned by time, on a very ſlender foundation; it probably extends no farther than may be owing owing to its gently diuretic quality, and the power of correcting a putrid tendency in the animal frame in common with most other vegetable productions.

The symptoms induced by *hemlock dropwort*, in the instance before us, sufficiently correspond with those recited in almost all the histories of its poisonous effects on record. Where the quantity swallowed was considerable, it did not fail, very soon, to bring on giddiness and sickness, which were succeeded by convulsions, and such a contraction of the jaw, as precluded, in many instances, the use of proper assistance. Those, who are disposed to see the history of this noxious vegetable farther illustrated, are referred to the following papers, among others that might be quoted.

*Philosoph. Trans. abridged.* Vol. II. p. 641.

*Phil. Trans. at large.* Vol. XLIV. p. 227, with an accurate engraving of the plant.

*Phil. Trans.* Vol. L. p. 856.

*Gent. Magazine*, 1747, p. 321, with a figure of the root copied from the foregoing.

*Gent. Magazine*, 1755, p. 114.

*London Med. Journal.* Vol. II. 1781, p. 40.

The most effectual method of attempting to relieve the unhappy sufferer in these melancholy cases, cafes, although obvious to all intelligent perfons, cannot be too often repeated—as foon as a miftake of this kind is difcovered, the moft inftant relief is to be expected by difcharging the poifon from the ftomach, without the leaft delay. To this end a large dofe of an emetic fhould be given. To a grown perfon, thirty or forty grains of ipecacuanha in powder, or an ounce and an half or two ounces of ipecacuanha wine; or fix, eight, or ten grains of emetic tartar; or twenty grains of white vitriol; affifting the operation, if poffible, by large draughts of diluting liquors, as warm water, thin gruel, which will probably be rendered more advantageous by the addition of a moderate quantity of vinegar, or other vegetable acid.

This is the firft and moft material ftep where vegetable poifons have been taken; and I clofe thefe hints by obferving, that vomiting is not lefs neceffary in the cafe of mineral poifons; fuch as *arfenic*, or *mercury fublimate*, *fugar of lead*, &c. unlefs they occafion of themfelves a fufficient difcharge this way. Here, however, great dilution is not lefs neceffary; but the addition of fome alkaline preparation to the diluting liquor is alfo ftrongly indicated, as a means of decompofing fuch fubftances, and rendering fuch

ſuch parts of them, as cannot be evacuated, in ſome meaſure inert. Moderate quantities, a tea ſpoonful, for inſtance, of ſalt of wormwood, of ſalt of tartar, or of potaſh, to a pint or a pint and an half of the liquid; and, as no time ought to be loſt, if none of the above are in readineſs, lye, made of wood-aſhes, will anſwer, only that the quantity muſt be much larger; as will ſoap-lye alſo. Even ſpirit of hartſhorn may contribute to the ſame purpoſe, if nothing elſe is immediately at hand. Should this evacuation be ſpeedily procured, time will be gained for ſuch farther means as the circumſtances of the caſe may require.

Blandford, May 29,
1784.

